# Facile fabrication of polyurethane/epoxy IPNs filled graphene aerogel with improved damping, thermal and mechanical properties†

**DOI:** 10.1039/c8ra04718a

**Published:** 2018-07-31

**Authors:** Chunmei Zhang, Yujie Chen, Hua Li, Hezhou Liu

**Affiliations:** State Key Laboratory of Metal Matrix Composites, School of Materials Science and Engineering, Shanghai Jiao Tong University Dongchuan Road No. 800 Shanghai 200240 China lih@sjtu.edu.cn; Collaborative Innovation Center for Advanced Ship and Deep-Sea Exploration, Shanghai Jiao Tong University China

## Abstract

In this article, polyurethane (PU)/epoxy (EP) interpenetrating polymer networks (IPNs) filled graphene aerogel (PEGA) was facilely fabricated by a one-step vacuum-assisted filling process. Effects of PU content on damping performance, thermal stability and mechanical properties of the PEGA composites were studied systematically. Results reveal that addition of graphene aerogel improves damping properties of PU/EP IPNs and increases the thermal decomposition temperature. Mechanical tests show that flexural strength, flexural modulus and Shore D hardness of the PEGA composites also improved by incorporation of graphene aerogel. The enhanced damping, thermal and mechanical properties of PEGA composites can be attributed to the uniform distribution of graphene sheets in the IPN matrix, which benefits from the three-dimensional interconnected porous network structure of the graphene aerogel used and good interfacial adhesion at the nanofiller-matrix interface. It is expected that the PEGA composites can be used as good structural damping materials in future.

## Abstract 2

PEGA composites were facilely fabricated by a one-step vacuum-assisted filling method, and exhibited improved damping, thermal and mechanical properties.

## Graphical abstract

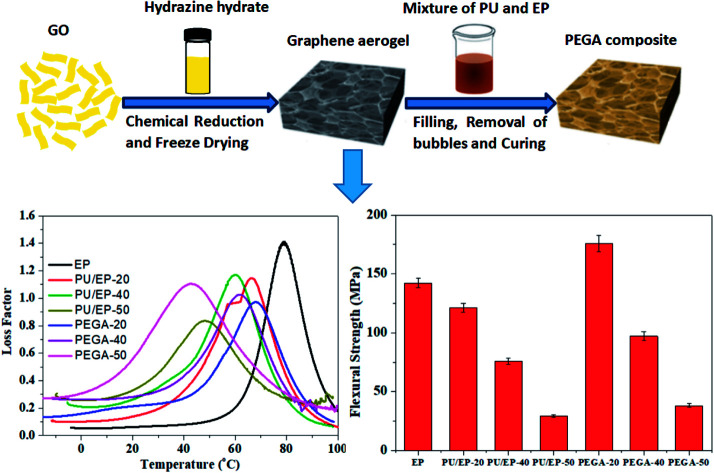

## 1. Introduction

Nowadays, due to rapid industrial development, noise and vibration pollution are becoming increasingly severe and exert adverse effects on human health, industrial safety and environment.^1–3^ In recent years, researchers have paid more attention to reducing noise and vibration in engineering fields.^4,5^ On account of their good viscoelasticity and processability, polymers are the most widely used damping materials as they can dissipate most external mechanical energy into heat through friction between macromolecular segments near *T*_g_.^6,7^

Loss factor (tan *δ*) is normally used to measure damping behavior of materials. The requirement for practical engineering materials is that the tan *δ* value should be above 0.3 and temperature range of tan *δ* > 0.3 should be as wide as possible.^8–10^ However, good damping performance of polymers is usually limited to a narrow temperature range of *T*_g_ ± 10 °C, which limits their practical use.^11^ Many techniques have been used to enhance damping properties of polymers, such as creation of polymer blends,^12,13^ copolymers,^14,15^ and interpenetrating polymer networks (IPNs).^8,16^ IPN is a novel type of polymer alloy comprising two or more crosslinked polymers held together by physical entanglement, and it is promising material with broad *T*_g_ ranges and excellent damping performance.^11^ Polyurethane (PU)/epoxy resin (EP) IPNs, which integrate the advantages of polyurethane's high damping performance and epoxy resin's high mechanical behavior, have been widely studied in previous reports,^9–11^ with expectations of obtaining good structural damping materials. However, the addition of PU into EP matrix can lead to reduction of mechanical and thermal properties due to the low modulus and thermal stability of PU polymer.

In the past few years, graphene sheets have been incorporated into a wide range of polymer matrices to enhance their thermal or mechanical properties, such as epoxy,^17,18^ polystyrene,^19^ polycarbonate,^20^ polyurethane,^21^ polyimide^22^ and polypropylene,^23^ and have achieved excellent results. The outstanding performance is attributed to the large specific surface area and exceptional thermal conductivity and Young's modulus of graphene sheets.^24^ The quality of nanofiller dispersion in the polymer matrix directly correlates with its effectiveness for improving mechanical, thermal, and other properties. One of the key problems for graphene applications is its poor dispersion in matrix because of the strong π–π interactions between graphene sheets, which can lead to inferior properties.^25,26^ Preparation of three-dimensional graphene aerogel, which is composed of individual graphene sheets, can effectively address aggregation problems. Many reports have studied applications of graphene aerogel in the fields of microwave absorption, batteries, supercapacitor, catalysis, *etc.*,^27–30^ and results demonstrate that three-dimensional graphene shows better properties compared to that of planar graphene sheets.

In this study, PU/EP IPNs filled graphene aerogel was fabricated by a simple one-step vacuum-assisted filling process. Moreover, its damping, thermal and mechanical properties were measured and the results and possible mechanisms were analyzed.

## 2. Experimental

### 2.1 Materials

Graphene oxide (GO) was prepared by a modified Hummers' method.^31^ The graphite powder used was bought from Qingdao Huatai Graphite Co., Ltd., China. Hydrazine hydrate (N_2_H_4_·H_2_O, AR), sodium nitrate (NaNO_3_, AR), potassium permanganate (KMnO_4_, AR), concentrated sulfuric acid (H_2_SO_4_, AR), hydrochloric acid (HCl, AR), and hydrogen peroxide (H_2_O_2_, AR) were purchased from Sinopharm Chemical Reagent Co., Ltd., China. All chemicals were used as received.

Double-pack polyurethane (PU) 130T-A (component A is isocyanate; B is polyalcohol) was purchased from Ausbond. E51, which was used as the epoxy monomer, and poly(ethylene glycol) diglycidyl ether (PEGGE), another epoxide acting as flexibilizer, were bought from Shanghai Resin Factory Co., Ltd., China. 4,4′-Diaminodiphenylmethane (DDM), a high temperature curing agent, Jeffamine D-400, a soft curing agent utilized to increase ductility, and the solvent acetone (CH_3_COCH_3_, AR) were supplied by Shanghai Macklin Biochemical Co., Ltd.

### 2.2 Preparation of graphene aerogel

Graphene aerogel was prepared by a simple one-step self-assembly process through hydrothermal reduction. First, a certain amount of graphite oxide (GO, 0.12 g) was added to 20 mL deionized water and stirred for 2 h to achieve a clear solution. Then, hydrazine hydrate (300 μL) was added dropwise into the above solution and stirred for several minutes. Following this, the mixture was sealed into a glass vial and placed in an oven at 95 °C for 12 h. Finally, the resulting hydrogel was washed with ethanol and deionized water several times to remove impurities and freeze-dried for 48 h to obtain graphene aerogel.

### 2.3 Fabrication of PU/EP filled graphene aerogel (PEGA)

**Fig. 1:**
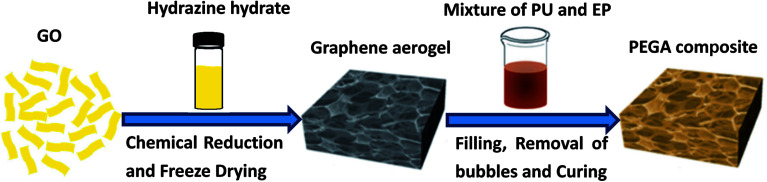
The schematic fabrication process of PEGA composite.

Epoxy E51 and polyalcohol were dried at 80 °C under vacuum for more than 6 h before use. A certain amount of curing agent DDM was dissolved in acetone and heated at 70 °C to obtain a clear solution. The schematic fabrication process of PEGA composite is shown in Fig. 1. In a typical procedure, 11.1% of the PU component A (PU-a) and 80% of E51 were dissolved in some amount of acetone and stirred for 60 minutes. Then 8.9% of the PU component B (PU-b) was added to the solution and stirred for another 60 minutes. Subsequently, certain amounts of PEGGE, Jeffamine D-400 and DDM were added into the above mixture and stirred for 30 minutes. Following this, the mixture was poured into the graphene aerogel and placed under vacuum at 80 °C for about 2 h to remove acetone and bubbles trapped in the composite. Finally, the composite was cured under 80 °C for 2 h and then 120 °C for 2 h to obtain PEGA-20 (mass ratio of PU/EP of 20/80). By changing the mass ratio of PU/EP to 40/60 and 50/50, composites PEGA-40 and PEGA-50 were obtained, respectively. The mass ratio of E51, PEGGE, DDM and D-400 in the composite was held constant at 9 : 1 : 1.45 : 0.73. Samples of pure EP, PU/EP-20, PU/EP-40 and PU/EP-50 were also prepared for comparative experiments. The calculated weight ratios of graphene aerogel in composites PEGA-20, PEGA-40 and PEGA-50 are 1.19, 1.16 and 1.15%, respectively.

### 2.4 Characterization and testing

X-ray diffraction (XRD) spectra were acquired by D/MAX2550/PC using Cu Kα radiation from 8° to 80° at a scan rate of 5°min^−1^ under 35 kV and 200 mA. X-ray photoelectron spectra (XPS) were recorded using a Kratos Axis Ultra DLD spectrometer. Raman spectra were taken on a SENTERRA R200 Raman spectrometer with 532 nm laser excitation. Nitrogen absorption and desorption measurements were performed with an Autosorb IQ instrument. Surface area was calculated by the Brunauer–Emmett–Teller (BET) method. Scanning electron microscopy (SEM) images were obtained on a Hitachi S-4800 field-emission SEM operated at 10 kV. Pyrolysis processes of the samples were studied using thermogravimetric analysis (TGA, PerkinElmer, Pyris 1 TGA) with a heating rate of 10 °C min^−1^ from room temperature to 800 °C under nitrogen atmosphere.

Dynamic mechanical measurements were performed on a DMA Q800 and rectangular specimens of 20 × 8 × 2 mm^3^ were used. Material property tests were conducted in tension mode at the frequency of 1 Hz. Temperature range was from −15 to 100 °C at a heating rate of 5 °C min^−1^, and storage modulus (*E*′), loss modulus (*E*′′), and loss factor (tan *δ*) values were obtained simultaneously. Flexural tests were performed with a three-point bending fixture according to ASTM D-790. Specimens of dimensions 50.8 × 12.7 × 3 mm^3^ were subjected to bending with a support span of 25.4 mm at a constant cross-head speed of 2 mm min^−1^ on a universal testing machine (BTC-T1-FR020 TN. A50, Zwick, Ger). Values were taken from an average of at least five specimens. Shore D hardness was measured according to DIN EN ISO 868 with a portable Shore D measuring instrument with specimens of 50 × 10 × 3 mm^3^ and reported results were an average of at least five measurements.

## 3. Results and discussion

**Fig. 2:**
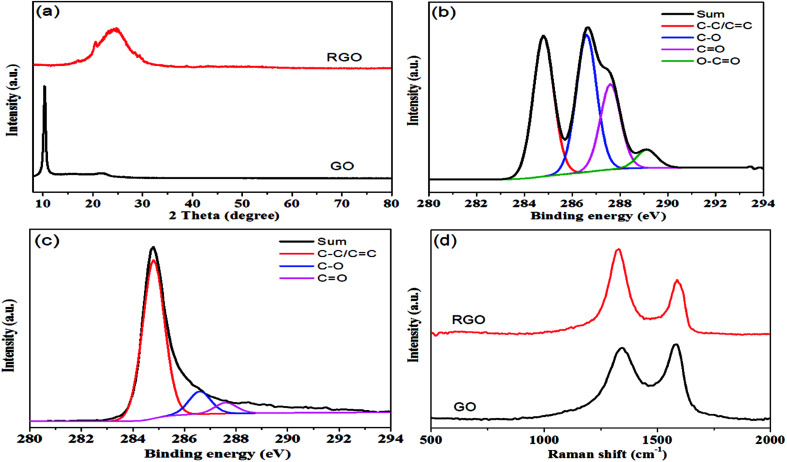
(a) XRD patterns of GO and RGO; (b) C1s XPS spectrum of GO; (c) C1s XPS spectrum of RGO; (d) Raman spectra of GO and RGO.

As shown in Fig. 1, hydrazine hydrate was used to reduce GO and after chemical reduction, graphene sheets, being hydrophobic, spontaneously self-assembled to form graphene aerogel. The reduction process was characterized by XRD patterns, XPS mappings and Raman spectra. The XRD pattern (Fig. 2(a)) of GO shows a diffraction peak at about 10.3°. After reduction, a relatively broad peak at about 25° can be found, indicating that GO has been reduced to graphene with fewer functional groups.^32,33^ The C1s XPS spectrum of GO is shown in Fig. 2(b), and the four different peaks at 284.8, 286.6, 287.6 and 289.1 eV can be attributed to C=C/C–C, C–O, C=O and O–C=O, respectively. After reduction, as shown in Fig. 2(c), it can be seen that the intensity of the peaks corresponding to oxygen-containing groups decreases drastically, proving that the reduction process was effective.^34^Fig. 2(d) shows the Raman spectra of GO and graphene aerogel. The D band peak can be observed at 1346 cm^−1^ and is associated with structural defects caused by the functional groups. The G band can be found at 1576 cm^−1^ and is the characteristic of sp^2^-hybridized carbon–carbon bonds.^35^ For GO, peak area ratio of the D band to the G band is 1.58, while the ratio is 2.10 for graphene sheets. According to previous reports, increase in the ratio of *A*(D)/*A*(G) indicates that numerous but smaller sp^2^ carbon domains were formed.^36^ The results of XRD, XPS and Raman spectra indicate that GO was effectively reduced to graphene sheets by hydrazine hydrate.

**Fig. 3:**
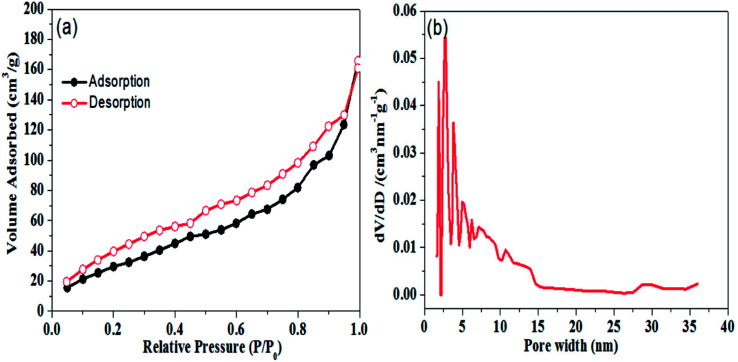
(a) Nitrogen adsorption and desorption isotherm and (b) pore size distribution plot of graphene aerogel.

Porous structure of the graphene aerogel was further validated by nitrogen physisorption measurements; results are shown in Fig. 3. In Fig. 3(a), it can be seen that N_2_ adsorption–desorption isotherms of graphene aerogel exhibit the Type II hysteresis loop, which is characteristic of pores of different sizes.^39,40^ BET surface area of the graphene aerogel was determined to be 122.6 m^2^ g^−1^. Moreover, pore size distribution of graphene aerogel calculated by DFT method indicates a large proportion of mesopores with size distribution from 2.5 to 20 nm with peak pore diameter of approximately 2.8 nm (Fig. 3(b)).

**Fig. 4:**
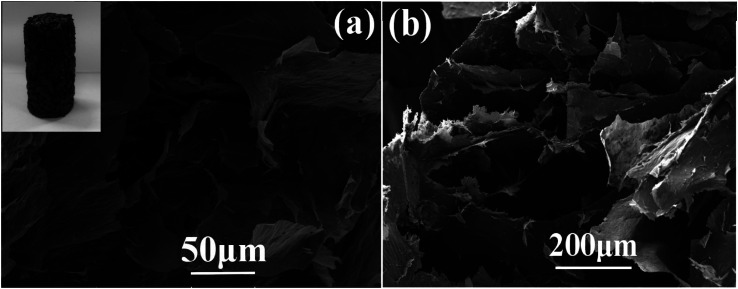
Photo (inset picture in (a)) and SEM images ((a) and (b)) of the prepared graphene aerogel.

**Fig. 5:**
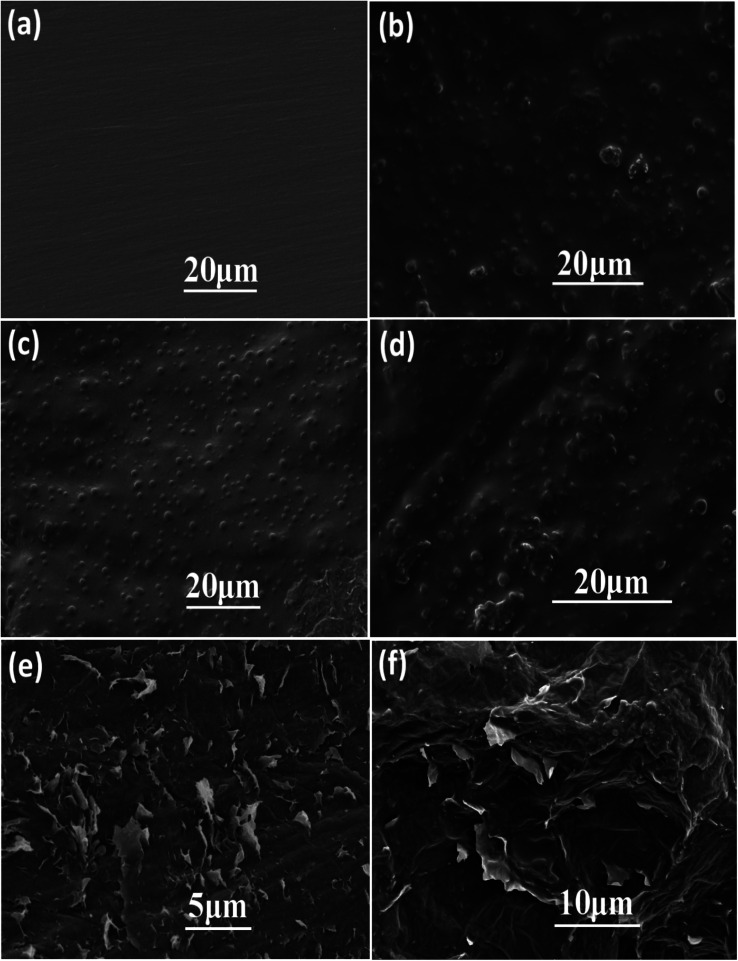
SEM images of (a) epoxy resin, (b) PU/EP-20, (c) PU/EP-40, (d) PU/EP-50, and (e) morphology and (f) fracture surface of composite PEGA-40.

Morphology and structure of the as-fabricated graphene aerogel were observed by scanning electron microscope, as shown in Fig. 4. The inset in Fig. 4(a) is a photo of the as-prepared graphene aerogel, and depending on the vessels used, it can be fabricated into specific sizes and shapes to meet the requirements in practical applications. From the SEM images in Fig. 4(a) and (b), it can be seen that the graphene aerogel exhibits a three-dimensional interconnected porous network structure, with pore sizes from approximately several micrometers to hundreds of micrometers. Fig. 5 and S1† show the surface morphologies of epoxy resin, PU/EP IPNs and PEGA composites, as detected by SEM analysis. Epoxy resin exhibits a single uniform phase, and after addition of PU polymer, PU/EP IPNs show a similar “sea-island” structure, in which epoxy resin is the continuous phase and spherical PU is the dispersed phase. The higher the PU prepolymer content, more spherical would be the PU particles. It was found that PU particles are relatively homogeneously distributed throughout the matrix with uniform particle size of approximately 1 μm. In addition, there is strong interfacial bonding between the two phases, which is very important for toughening. Moreover, there is a certain phase interface between the two phases, which indicates that the two phases do not constitute a molecular interpenetrating network structure, and this is a necessary condition to obtain good damping properties.^37^

The fractured surface morphologies of epoxy resin, PU/EP IPNs and PEGA composites were also analyzed by SEM, as shown in Fig. 5 and S1.† It can be seen that pure EP has characteristic brittle features with smooth fractured surfaces. With the addition of PU, the fractured surfaces of PU/EP IPNs become rougher and more uneven, indicative of ductile nature of the fracture.^16,38^ PEGA composites were fabricated by filling the graphene aerogel with PU/EP IPNs. The images clearly demonstrate that graphene nanosheets are well-dispersed in the PU/EP matrix and they distribute as a three-dimensional network through the polymer matrix. In Fig. 5(f), it can be observed that the PEGA composites possess rough fractured surfaces and the majority of the protruding graphene nanoplatelets are coated by PU/EP polymer, which can be attributed to strong interfacial adhesion and good compatibility between the polymer matrix and graphene sheets.^26^ From the SEM images of graphene aerogel in Fig. 4, it can be seen that the graphene sheets possess wrinkled (rough) surfaces, which can enhance nanofiller-matrix adhesion through interlocking effect, as previously reported.^17,18,45,46,50^ In addition, residual oxygen-containing groups on the surface of RGO sheets can form hydrogen bonds or react with polymer chains during the high-temperature curing process, which is beneficial for interfacial adhesion between nanofiller and polymer matrix.^47–50^ Such strong interactions are favorable for stress transfer from the polymer matrix to the graphene sheets, leading to improvement in mechanical properties of the composites compared to those of pure PU/EP IPNs.

**Fig. 6:**
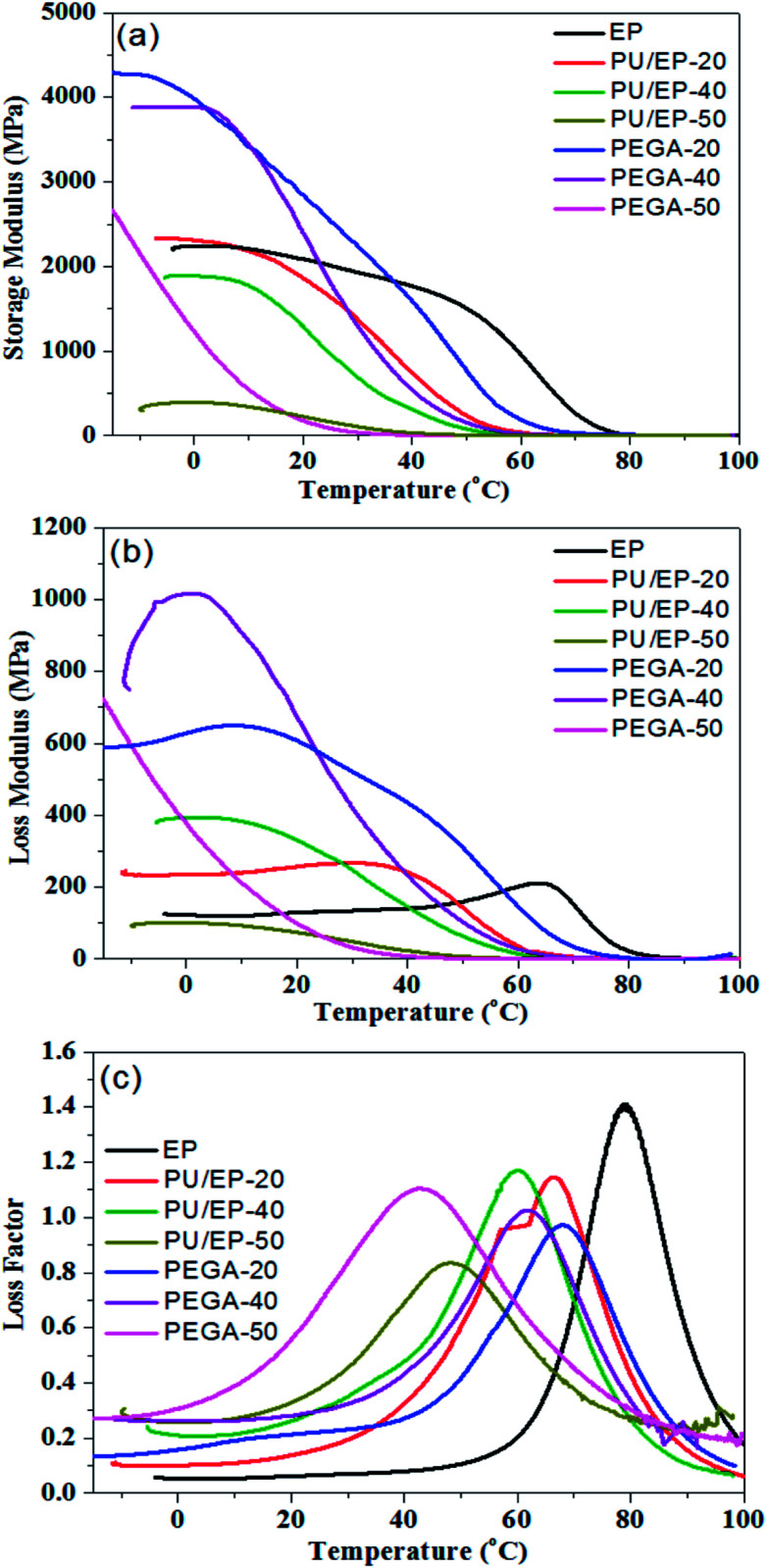
Variation plots of (a) storage modulus, (b) loss modulus and (c) loss factor as a function of temperature for epoxy resin, PU/EP IPNs and fabricated PEGA composites at 1 Hz.

PEGAs were fabricated by filling the graphene aerogel with PU/EP mixture. The damping properties of the epoxy resin, PU/EP IPNs and PEGA composites were investigated by dynamic mechanical analyzer (DMA) and the parameters of storage modulus (*E*′), loss modulus (*E*′′) and loss factor (tan *δ*) of materials were recorded simultaneously. Fig. 6(a) shows the variation curves of different materials' *E*′ values as a function of temperature. Storage modulus is an important property to assess load bearing capacity of a material and hence, high *E*′ value indicates high stiffness of material.^4,5^ It was found that *E*′ values of PU/EP IPNs are all lower than those of the epoxy resin and gradually decreased with increasing PU amount. As shown in Table 1, *E*′ values of epoxy resin, PU/EP-20, PU/EP-40 and PU/EP-50 are 2074.9, 1862.9, 1292.0 and 225.9 MPa, respectively. After addition of graphene aerogel, storage modulus values of composites PEGA-20 and PEGA-40 were improved compared with those of the corresponding PU/EP IPNs and their *E*′ values were higher than that of epoxy resin near room temperature. This significant enhancement in mechanical performance of PEGAs can be attributed to the good dispersion of graphene sheets in the composites, which benefits from the three-dimensional network structure of graphene aerogel and the good interaction between graphene sheets and polymer matrix, which could be caused by the reaction between residual oxygen-containing functional groups of graphene sheets and the PU/EP mixture during the curing process.^18,19^ As shown in Table 1, the storage modulus values of PEGA-20, PEGA-40 and PEGA-50 at 20 °C are 2845.7, 2382.7 and 176.6 MPa, respectively.

**Table 1:** Damping properties of different materials at 1 Hz

Sample	Storage modulus (*E*′) (MPa) at 20 °C	Loss modulus (*E*′′) (MPa) at 20 °C	Loss factor (tan *δ*) at 20 °C	*T*_g_ (°C)	Temperature range (°C)/tan *δ* > 0.3 (Δ*T*)
Epoxy resin	2074.9	129.6	0.062	78.9	63.6–95.9 (32.3)
PU/EP-20	1862.9	257.0	0.138	65.4	38.6–83.4 (44.8)
PU/EP-40	1292.0	338.8	0.262	60.0	24.6–78.9 (54.3)
PU/EP-50	225.9	74.5	0.33	48.4	16.2–76.2 (60.0)
PEGA-20	2845.7	606.5	0.213	67.9	43.3–85.7 (42.4)
PEGA-40	2382.7	670.1	0.281	61.8	25.5–81.2 (55.7)
PEGA-50	176.6	98.6	0.56	42.7	−1.3 to 80.4 (81.7)

Fig. 6(b) shows the loss modulus of epoxy resin, PU/EP IPNs and PEGA composites as a function of temperature. Loss modulus (*E*′′) is a measure of energy dissipated as heat per unit cycle under mechanical deformation and it is used to characterize viscosity of a material.^1,8^ It is evident that all the as-fabricated PEGAs show higher *E*′′ values than corresponding PU/EP IPNs, which indicates that PEGA composites could dissipate more mechanical vibration and noise into heat energy. The composite PEGA-40 displays the best loss modulus behavior at room temperature and its *E*′′ value represents an increase of about 977.9% compared to that of PU/EP-40 at the same temperature, which may be due to the graphene aerogel generating a high filler boundary sliding (filler–filler) and interfacial sliding (filler-matrix) under external dynamic loading, which dissipates more mechanical energy into heat.^2,16^

Loss factor (tan *δ*) is defined as the ratio of storage modulus and loss modulus. Higher tan *δ* values indicate better energy dissipation capabilities of materials.^39^ Normally, service environment for most engineering damping materials is near room temperature; hence, the value of tan *δ* should be high and the temperature range of tan *δ* > 0.3 should be as wide as possible near room temperature.^37^ Loss factor values of epoxy matrix, PU/EP IPNs and PEGA composites as a function of temperature at 1 Hz are shown in Fig. 6(c). It is evident that loss factors of all PU/EP IPNs were greatly enhanced compared with those of epoxy matrix and gradually improved with the increase in PU content. Moreover, damping loss factors of PEGA composites are higher than those of corresponding PU/EP IPNs. For composite PEGA-20, the damping loss factor (tan *δ*) was 0.213 at room temperature and the temperature range of tan *δ* > 0.3 was from 43.3 to 85.7 °C. For PEGA-40, the tan *δ* value is 0.281 at room temperature and the temperature range of tan *δ* > 0.3 was from 25.5 to 81.2 °C, which covers the usual applied temperature range. Composite PEGA-50 exhibits the best tan *δ* value of 0.56 and the widest temperature range of tan *δ* > 0.3 of 81.7 °C from −1.3 to 80.4 °C. These results indicate that damping performance of PEGA composites are gradually improved with the increase in PU content.

*T*_g_ value in this manuscript is defined by the temperature at the peak of the energy dissipation curves (tan *δ* curves), as reported in other studies.^18,19,40^ It can be observed that all the glass transition temperatures of PU/EP IPNs shift to lower temperatures and decrease with the increase in PU weight ratio due to addition of low *T*_g_ PU polymer. Furthermore, glass transition temperatures of composites PEGA-20 and PEGA-40 improved compared with those of corresponding PU/EP IPNs, while *T*_g_ of composite PEGA-50 is lower than that of PU/EP-50. In the PEGA composites, two possible factors influence their glass transition temperatures. On one hand, the added graphene aerogel can absorb the nitrogen-containing curing agent or isocyanate chains of PU, which can disturb the stoichiometric reaction of the polymers and thus lead to reduction of *T*_g_ by decreasing the crossing-linking density of the system. On the other hand, the wrinkled structure and higher stiffness of graphene sheets can constrain molecular mobility of the polymer chains in the substantial interphase zone around the nanosheets. Moreover, 3D graphene aerogel can provide higher surface area with more interphase zone in contact with the polymer matrix and thus shift *T*_g_ to higher temperature. The final effect on *T*_g_ will depend on the balance of these two effects, namely, influence on reaction conversion and molecular confinement, as reported by other studies.^40,46,49,50^ For composite PEGA-50, *T*_g_ decrease may be attributed to reduction of cross-linking density of the polymers functioning more during the complicated reaction system. As a result, reduced cross-linking density of the polymers leads to lower storage modulus of composite PEGA-50 when compared with that of PU/EP-50.

**Fig. 7:**
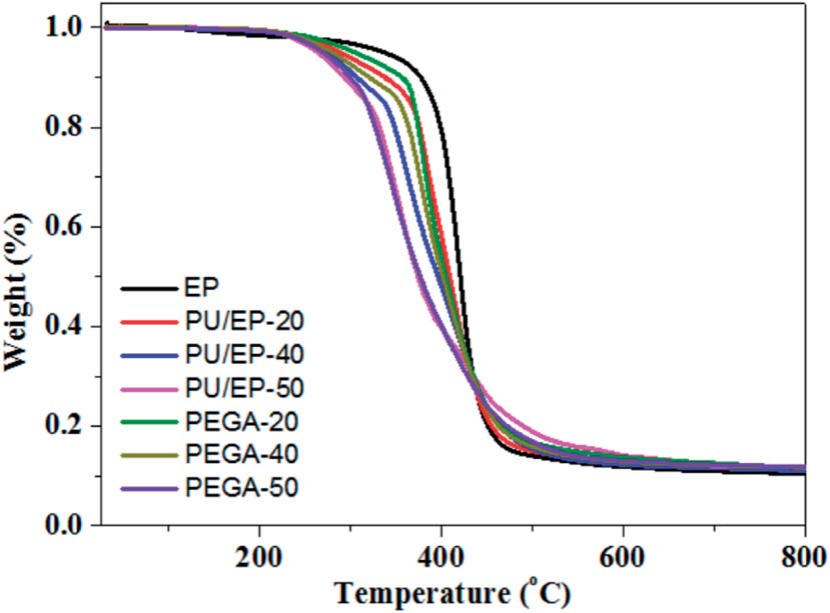
TGA curves of the prepared epoxy resin, PU/EP IPNs and PEGA composites in N_2_ atmosphere.

TGA was used to assess thermal stability of the epoxy resin, PU/EP IPNs and PEGA composites; their thermograms are presented in Fig. 7. Characteristic temperatures of samples in the pyrolysis process are listed in Table 2. As shown in Fig. 7, a sharp decrease in weight loss between 300 and 450 °C for the as-prepared materials can be observed, which is ascribed to decomposition of polyurethane and epoxy resin polymer, as reported in other studies.^16^ In addition, it can be seen that the thermal stability of PU/EP IPNs is much lower than that of neat epoxy resin, which is due to the lower thermal stability of polyurethane.^10,16^ After the addition of graphene aerogel, the thermal stability of all the PEGA composites improved significantly compared with those of corresponding PU/EP IPNs. As shown in Table 2, decomposition temperatures *T*_5_ (5% weight loss) of PEGA-20, PEGA-40 and PEGA-50 increased by 15.9, 6.3, and 6 °C, respectively, compared to corresponding IPN polymers. In addition, *T*_10_ of PEGA-20, PEGA-40 and PEGA-50 increased by 18.1, 13.7, and 6.7 °C, respectively. Moreover, residual weights at 700 °C of PEGA composites also increased compared to those of corresponding PU/EP IPNs. These results clearly indicate that introduction of graphene aerogel into PU/EP IPNs greatly enhanced thermal stability of the composites. The increase in thermal stability with addition of graphene sheets may be attributed to the higher heat capacity of graphene sheets compared to polymer matrix and a better barrier effect of graphene sheets, which retards the volatilization of polymer decomposition products due to the good dispersion and interface in PU/EP IPNs.^41^ In addition, the increased char residue can form a char barrier to protect the nanocomposite surface from oxygen and a mass and heat barrier to enhance the thermal stability.^42^ Similar phenomena have been reported in earlier studies, showing that addition of graphene sheets can improve thermal stability of the resulting composites.^41,42^

**Table 2:** Characteristic temperatures of samples in thermal degradation process

Samples	*T*_5_ a (°C)	*T*_10_ b (°C)	*T*_15_ c (°C)	*T*_60_ d (°C)	Residual weights at 700 °C (%)
Epoxy resin	335.8	377.0	391.1	426.7	10.0
PU/EP-20	288.0	337.8	365.8	419.9	11.9
PU/EP-40	272.8	306.7	337.1	414.9	11.6
PU/EP-50	263.0	292.8	317.9	398.8	11.9
PEGA-20	303.9	355.9	369.4	418.8	12.6
PEGA-40	279.1	320.4	353.2	416.1	12.3
PEGA-50	269.0	299.5	317.4	400.4	12.1

a *T*_5_, temperature of 5% weight loss.

b *T*_10_, temperature of 10% weight loss.

c *T*_15_, temperature of 15% weight loss.

d *T*_60_, temperature of 60% weight loss.

**Fig. 8:**
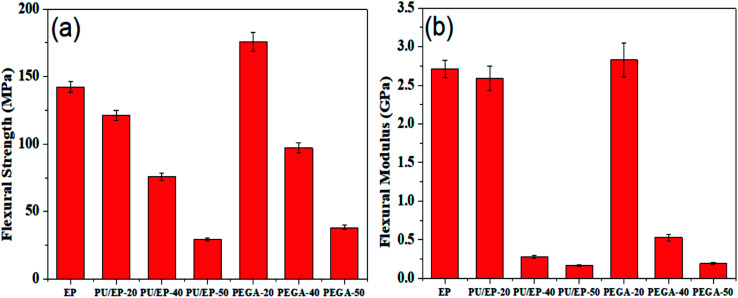
Variation plots of (a) flexural strength and (b) flexural modulus of the prepared epoxy resin, PU/EP IPNs and PEGA composites.

Given the excellent elastic modulus (∼1100 GPa) and intrinsic strength (125 GPa) of graphene sheets,^24^ we examined the effect of graphene sheets on the mechanical properties of PU/EP IPNs. Flexural strength and flexural modulus of the as-fabricated materials are shown in Fig. 8(a) and (b), respectively. It can be observed that PU/EP IPNs possess lower flexural strength and flexural modulus compared with those of epoxy resin. Also, with the increase in PU content, flexural strength and flexural modulus values of PU/EP IPNs decrease gradually. Physical entanglement of polyurethane and epoxy resin in the interpenetrating polymer networks is beneficial for improvement of mechanical properties. However, when content of PU components reaches a certain value, compatibility between the network structures of PU and EP deteriorates and the system undergoes phase separation. Moreover, the glass transition temperature of polyurethane is usually in the sub-zero range due to its soft segments; thus, it exhibits lower mechanical and thermal properties compared to epoxy resin. Due to the above two reasons, PU/EP IPNs exhibit reduced mechanical and thermal properties and with the increase in PU content, the PU/EP IPNs exhibit a decreased tendency.^51–53^ After addition of graphene aerogel, flexural strength and flexural modulus of all PEGAs improved compared with those of corresponding PU/EP IPNs. For PEGA-20, flexural strength and flexural modulus significantly improved, with values of 175.8 MPa and 2.83 GPa, exhibiting an increase of about 45.1 and 9.27%, respectively, compared with PU/EP-20, and the values are higher than those for epoxy resin. For composite PEGA-40, flexural strength significantly improved from 75.9 to 97.1 MPa and flexural modulus greatly enhanced from 0.28 to 0.53 GPa compared with PU/EP-40 IPN, showing an increase of about 27.9 and 89.3%, respectively. There are several possible reasons for the enhancement of mechanical performance of PEGAs. The three-dimensional porous network structure of graphene aerogel guarantees uniform distribution of graphene sheets in the matrix and also easily forms mechanical percolated networks in the composites, which is beneficial for stress transfer from the polymer matrix to the graphene sheets, thus leading to the improvement in the mechanical behavior of the PEGA composites compared to that of the PU/EP IPNs.^21–23^ In addition, graphene sheets have numerous residual oxygen-containing surface groups which can form hydrogen bonds or react with PU/EP mixture during the curing process, resulting in good interfacial adhesion at the nanofiller-matrix interface.^19,26^ Moreover, the two-dimensional geometry and large aspect ratio of graphene platelets may benefit the enhancement of composites' mechanical behavior.^24^

**Fig. 9:**
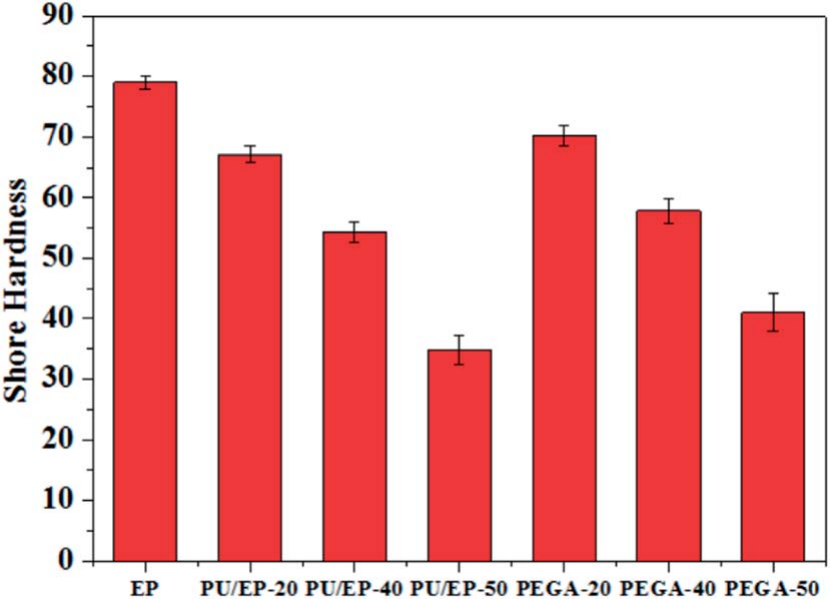
Variation plots of Shore hardness of the prepared epoxy resin, PU/EP IPNs and PEGA composites.

As shown in Fig. 9, with the addition of graphene sheets, the PEGA composites show improved hardness compared with that of corresponding PU/EP IPNs. The hardness decreases with the increase in PU content due to low hardness of the PU polymer. Shore D hardness values of PEGA-20, PEGA-40 and PEGA-50 increase by about 4.62, 6.45 and 17.8%, respectively, compared with those of PU/EP-20, PU/EP-40 and PU/EP-50 IPNs. It is well known that conventional hardness is a measure of resistance of the material to local deformation under nearly static conditions^43^ and these results indicate that PEGA composites could possess a higher wear resistance.^44^

## 4. Conclusions

In this study, polyurethane (PU)/epoxy (EP) interpenetrating polymer networks (IPNs) filled graphene aerogel (PEGA) were facilely fabricated by a one-step vacuum-assisted filling process. The effects of PU content on damping performance, thermal stability and mechanical properties of the PEGA composites were studied systematically. Composite PEGA-20 (weight ratio of PU/EP of 20/80) exhibits a damping loss factor (tan *δ*) of 0.213 at room temperature and the temperature range of tan *δ* > 0.3 from 43.3 to 85.7 °C. PEGA-40 has tan *δ* value of 0.281 at room temperature and a temperature range of tan *δ* > 0.3 from 25.5 to 81.2 °C, which covers the usual applied temperature range. Thermal decomposition temperatures *T*_10_ (10% weight loss) of PEGA-20 and PEGA-40 increase by about 18.1 and 13.7 °C compared with those of corresponding PU/EP-20 and PU/EP-40. Furthermore, flexural strength, flexural modulus and Shore D hardness of composite PEGA-20 increased by approximately 45.1, 9.27 and 4.62%, respectively, while the values for PEGA-40 increased by 27.9, 89.3 and 6.45%%, respectively, when compared with those of corresponding PU/EP IPNs. The enhanced damping, thermal and mechanical properties of PEGA composites can be attributed to the uniform distribution of graphene sheets in the IPN matrix, which benefits from the three-dimensional interconnected porous network structure of graphene aerogel used and good interfacial adhesion at the nanofiller-matrix interface. It is expected that the PEGA composites can be used as good structural damping materials in future.

## Conflicts of interest

There are no conflicts to declare.

## Supplementary Material

RA-008-C8RA04718A-s001
