# Supplementary material for: Facile fabrication of polyurethane/epoxy IPNs filled graphene aerogel with improved damping, thermal and mechanical properties
Source: RSC Adv. 2018 Jul 31;8(48):27390–9. doi: 10.1039/c8ra04718a (PMC9083253; doi:10.1039/c8ra04718a)
Supplement: RA-008-C8RA04718A-s001 [file RA-008-C8RA04718A-s001.pdf]

Electronic Supplementary Material (ESI) for RSC Advances.
This journal is © The Royal Society of Chemistry 2018

Supporting Information

Facile fabrication of polyurethane/epoxy IPNs filled graphene aerogel with improved damping, thermal and mechanical properties

Chunmei Zhang[a,1], Yujie Chen[a,1], Hua Li[a,b,*] and Hezhou Liu[a,b]

[a]State Key Laboratory of Metal Matrix Composites, School of Materials Science and Engineering, Shanghai Jiao Tong University, Dongchuan Road No. 800, Shanghai 200240, China.

[b]Collaborative Innovation Center for Advanced Ship and deep-Sea Exploration, Shanghai Jiao Tong University.

*Corresponding author: Hua Li (email: lih@sjtu.edu.cn)

[1]These authors contributed equally to this work.

Figure S1 The SEM images of the fracture surface of (a) epoxy resin, (b) PU/EP-20, (c) PU/EP-40, (d) PU/EP-50; (e), (f) and (g) different magnifications of the morphology of composite PEGA-40; and (h) the morphology of PU/EP-40
